# Information technology challenges in a public hospital during the COVID-19 pandemic

**DOI:** 10.6061/clinics/2021/e2648

**Published:** 2021-02-16

**Authors:** Jorge Futoshi Yamamoto, Izabel Oliva Marcílio de Souza, Leila Suemi Harima Letaif, Vilson Cobello-Júnior

**Affiliations:** I Nucleo Especializado de Tecnologia de Informacao (NETI), Hospital das Clinicas HCFMUSP, Faculdade de Medicina, Universidade de Sao Paulo, Sao Paulo, SP, BR; II Hospital das Clinicas HCFMUSP, Faculdade de Medicina, Universidade de Sao Paulo, Sao Paulo, SP, BR

In the past few decades, academic healthcare centers have positively impacted medical care quality by establishing well-structured information technology (IT) departments to address medical and research issues globally (1,3). However, information technology is not a panacea to solve all problems concerning healthcare; many aspects of applying IT need to be assessed, such as safety, organization, and environment (6). Nevertheless, IT possesses great potential for creatively resolving and answering the challenges that risky situations pose to society.

The COVID-19 pandemic is an extraordinary challenge since, unlike the Spanish flu pandemic in 1918, which took approximately a year to spread worldwide, its infection disseminated in a few weeks (5). COVID-19, an acute viral infection caused by the novel coronavirus (SARS-CoV-2), was initially detected in Wuhan, China, in December 2019. Due to the virus's high transmissibility and the severity of the disease, the World Health Organization (WHO) declared the COVID-19 outbreak a public health emergency of international concern under the International Health Regulations (2005) (8).

Healthcare systems around the world utilized various resources, such as science and technology, in preparation to handle the COVID-19 pandemic. Fortunately, several digital technologies that assisted in tackling COVID-19 infections were mature enough at the start of 2020 to be applied to the field of medicine. Ting et al. (12) divided these digital technologies into four groups: Internet of Things (IoT), big data analytics, artificial intelligence (AI), and blockchain technology.

The Hospital das Clínicas da Faculdade de Medicina da Universidade de São Paulo (HCFMUSP)—the most extensive university-affiliated hospital in São Paulo City, Brazil—played an essential role in diagnosing and treating the highly complex COVID-19 cases. Since the end of March 2020, the hospital has reserved one of its largest buildings—with 500 beds and 300 adult intensive care unit (ICU) beds—to treat COVID-19 patients exclusively. Furthermore, all of the hospital’s sectors were utilized to deal with COVID-19 patients.

According to Ting et al. (12), the HCFMUSP utilized two of the aforementioned four digital technology groups, namely IoT and AI, in addition to telemedicine. Several departments were integral to effectively applying these resources: the IT department, Núcleo Especializado de Tecnologia de Informação (NETI), which manages the central administration systems and electronic health records; the Clinical Engineering Department, which provides and maintains the highly technological facilities and operational services at the hospital; the Clinical Director’s Office, which promotes the development of health actions and services, supports professional training, and strives for comprehensive patient care; and lastly, the Crisis Management Committee, which was mobilized to assist with strategic planning (7,11,13).

## TECHNOLOGIES APPLIED AT THE HOSPITAL

Among their various statements, the WHO^1^ proposed the adoption of certain preventive measures against the virus, such as social distancing and staying at home (shelter-in-place). Consequently, traditional medical consultations were deemed risk factors.

Technological resources that allowed for remote medical practice were used to solve this problem. Keesara et al. (5) commented that digital technologies have existed for decades, but that they did not exhibit extensive infiltration in the market due to heavy regulations, high costs, and shortages in technical support.

## NETWORK INFRASTRUCTURE

Virtually all digital technologies require a network connection. Therefore, the existing network infrastructure at the hospital came under intense scrutiny. Consequently, to support the implementation of digital technology, investments to combat the COVID-19 pandemic had to be diverted to expenses concerning network communication and electrical infrastructures.

Fortunately, the hospital had installed a metropolitan network backbone meant to provide fast and reliable access to several available information systems in 2018. In turn, each institute should provide a local network infrastructure to connect to the main backbone. However, due to the space reserved for COVID-19 patients, the network needed to be reinforced, mainly with a focus on wireless access. In a joint effort with several sponsors, the hospital managed to set up a network on an emergency basis to supply the necessary connections.

## RESCHEDULING MEDICAL APPOINTMENT

Using digital technologies was critical in facilitating communication with those patients and families who were long-term clients of large health systems (10). Assigning the largest hospital building exclusively for COVID-19 patients, in combination with the social distancing and shelter-in-place protocols, necessitated extensive alterations to the scheduled consultations. All of the existing consultations scheduled for the first half of 2020 needed to be swiftly changed.

The NETI developed an application utilizing voice recognition technology that listed scheduled patients’ consultations and phone numbers. The application screened patients that were available for phone calls, and subsequently connected them with a physician to reschedule. We found that, for cultural reasons, most patients preferred direct conversations with their physicians; confidence in automatic voice services is lacking, especially among low-income patients. Therefore, it is crucial to engage patients directly during the rescheduling process.

**Figure 1:**
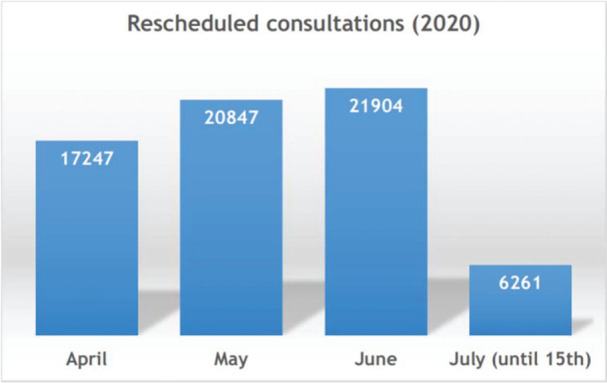
Rescheduled medical consultations (2020) via the application.

Between April 1^st^ and July 15^th^, 66,259 rescheduling activities were facilitated through the application (Figure 1).

## BED MAPPING

Unfortunately, the planning and management concerning bed capacities had to be evaluated within an environment of uncertainty, variability, and limited resources (4). With regard to the COVID-19 pandemic, above factors seem incredibly unfavorable.

The hospital reserved 500 beds and 300 ICU beds to accommodate the COVID-19 patients. Although the hospital already possessed a bed mapping web system, it was necessary to adapt it for this situation. The web system displays a dashboard panel which allow staff to monitor the occupied beds. Initially, the panel displayed limited data, such as patient identification; however, after the modifications made by NETI, the dashboards provided extensive information on the patients.

Between March 30^th^ and August 11^th^, 2020, the average number of patients admitted daily was approximately 30.3, with a maximum of 56. Given this data, the need for bed mapping became apparent; moreover, we had to account for and control an aggravating factor: nosocomial infections occurred due to COVID-19’s high virulence. For example, after a patient was discharged, the bed that they had occupied had to immediately undergo a sterilization process.

## SUPPORT FOR CLINICAL STAFF

Due to the high virulence of COVID-19, all medical teams must continuously assess their health and look for any symptoms. Similarly, other hospital employees who were not in direct contact with patients also needed to be vigilant of their symptoms.

The Centro de Atendimento ao Colaborador (CEAC)—the hospital medical employee center—has been altered to deal solely with suspected COVID-19 cases since March 2020. To prevent the hospital team from dealing with crowding situations related medical support, NETI created an application that monitors the number of people in line for medical care. The center’s concierge counts the number of people and records these values in the system. If the number is high, people can postpone their medical support visit until the number goes down. Additionally, the system calculates the expected waiting time based on the registered flux of people.

## TELEPRESENCE

All COVID-19 patients were strictly isolated inside the hospital. To avoid further contagion, their relatives and friends were forbidden from coming to the hospital and, subsequently, faced difficulties in getting news from their loved ones. Considering the complications of face-to-face communication during this pandemic, the HCFMUSP incorporated telepresence communication into its work routine, with the aim of bringing patients and the healthcare team closer to their families.

Additionally, with the adoption of remotely controlled robots, cross-contagion among the assistance teams as well as the amount of required personal protective equipment can be substantially reduced. Technically, the robot is only an adapted Apple iPad mounted on a remote-controlled propulsion system. The improvements made concerning the wireless network structure proved essential to these robots; they communicate through Wi-Fi. 

Although telemedicine refers to relatively old technology (9), the Federal Ministry of Health has only started regulating its practice under emergency conditions this year. The HCFMUSP established a telemedicine network to support clinical decisions for care patients and train several public hospitals connected to its network (2). Expert intensive care physicians from the HCFMUSP remotely trained frontline healthcare professionals through the telemedicine network.

## AI AND IMAGE ANALYSIS

The hospital’s Instituto de Radiologia (Radiology Institute) launched an important initiative for developing deep learning algorithms that analyze X-ray and computerized tomography (CT) images. The project, named RadVid-19, resulted from a partnership with private companies.

The RadVid-19^2^ project aims to provide specialized support to hospitals in all regions of the country through AI analysis reports. Additionally, it offers a second opinion channel for interpreting CT and X-ray examinations of patients with positive or suspected diagnoses of COVID-19. The examinations and data obtained during the analyses will also aid the construction of a database concerning positive cases, which can support studies and research to combat the pandemic.

## CONCLUSION

The COVID-19 pandemic brought the medical activities that take place inside a hospital under scrutiny. Drastic changes had to be made since even the face-to-face interaction between clinical staff and patients became hazardous due to the risk of contagion.

According to the examples above, IT hospital groups support the fight against the COVID-19 pandemic to a great extent. Combating the pandemic through the support of technological resources has made numerous contributions to medical treatments, many of which the hospital will thoroughly incorporate.

## AUTHOR CONTRIBUTIONS

Ragazzo L contributed in article’s conception, researcher, manuscript development and accountability. Puech-Leao P and de Luccia N contributed in critical revision and approval of the manuscript. Wolosker N contributed in article’s conception and critical review. Saes G contributed in researcher and critical revision. Ritti-Dias RM and Kamikava DY contributed in researcher and statistical analysis. Kanikava D participated as researcher. Zerati AE contributed in article’s conception, critical review and approval of the manuscript.
